# Dinner of the General Practitioners

**Published:** 1830-04-01

**Authors:** 


### XXXVII.

### Dinner of the General Practitioners.

We learn that a considerable number of the general practitioners of this metropolis mean to celebrate the late decision of Lord Tenterden, by a public dinner early in March. We, who are their friends, recommend to them temperance—not as regards turtle soup or the Tuscan grape—but as regards the expression of their sentiments and the adoption of their resolutions. They meet for a public good—one that may be equally beneficial to the community at large, and to themselves, as a great branch of the medical profession; but, as they will experience more difficulties in attaining their object than they are yet aware of, prudence as well as good taste ought to restrain them from exuberant exultation, and, above all, from the remotest approximation to the introduction of medical politics or party feelings. The subject which draws them together, and which will naturally excite opinions or even discussions, has no necessary connexion with medical politics, and whoever introduces topics that can, in the slightest degree, excite the jealousy, or hurt the feelings of any other branch of the profession, is no friend to the object in pursuit, and will infallibly injure the cause in which they are embarked. The profession has been too long torn by dissensions that tend to lower it in the eyes of the world at large—and such a meeting as that in question, should endeavour to heal the wounds of medical society, rather than open them out afresh. It is only by unanimity that the general practitioners can hope to attain the object of their solicitude—

but that unanimity among themselves will be best cemented by friendly feelings to their brethren of every description.

They will be wise not to aim at too much in the outset. No great and lasting reformation, in our profession, or in any other, was ever effected PER SALTUM. There is a natural reaction against all sudden and violent revulsions or innovations in society—and no unanimity can be expected under such circumstances. We have heard that it it is proposed that the general practitioners should relinquish pharmacy in toto, and practise merely by prescription and fees. We have very little doubt that the rapidly enlarging scale of medical education will ultimately bring the practice into this line; but we humbly suggest that an intermediate step would be most prudent, in the first instance:—namely, a reduction of the quantum of medicines dispensed, and an equivalent in charges for attendance.* There is nothing to prevent the adoption of this plan, except want of unanimity. There is no law necessary in the case, nor will the public be long averse to this procedure, after the beneficial influence of it is fully and calmly explained to them by an energetic but dispassionate address from the general practitioners themselves. This address should be carefully and cautiously drawn up, and it should be widely circulated through the medium of the public press. The framing of this address, indeed, appears to be the very first step they should take; and a subscription ought to be immediately raised for defraying the expenses of it. Meetings of the practitioners themselves should be held in all the towns of the empire, and resolutions entered into to support the principle of the address.

Once more we conjure the practitioners who meet at dinner on this occasion to avoid intemperate sallies of feeling, and all appearance of exultation respecting the recent decision in their favour. The public is a capricious and inconsistent, as well as inconstant monster which is readily excited—easily led astray—and managed with great difficulty, not to say DANGER. A convivial meeting is a bad *legislative* assembly:—and sentiments may be broached by well meaning but wrong-headed individuals, under the influence of ardent feelings, which may do more injury to the cause for which they are congregated than they may imagine. If any unwise exultation be displayed on this occasion, it is not improbable that the public may take alarm, and construe the celebration of an event, in reality beneficial to themselves, into a victory gained over them by the general practitioners.* These cautious considerations will be looked upon by the young and sanguine, no doubt, as the timid fears of grey hairs; but the whole history of mankind teaches us,

---

* There is a large class of people now, and there ever will be one, who are unable to pay for more than even a small quantity of medicine. What would become of these, were the general practitioner to give up entirely the dispensing of medicine? They would be thrown into the hands of chemists and druggists! This is a class which, though yielding little profit individually to the medical practitioner, return a considerable annual income in the aggregate. It is generally among this class, too, that the reputation of the young practitioner is foundationed, and his future fame and fortune secured. It will not be prudent to leave such a class only the alternatives of physicking themselves or going to a dispensary.

* Nothing could be more injudicious than the wording of the advertisement for this dinner. A "victory"— a "triumph!" This augurs ill—and we venture to predict, that if the men who penned such imprudent notices bear sway at the dinner, the whole will end, as did the meetings at the Freemasons' Tavern, in destruction to the object in view. Let the company only assume the appearance of a party, and there is an end of the affair. Every respectable practitioner will wash his hands of the procedure.

that the more meekly we bear our triumphs the more solid will be our victories.

---

XXXVIII.

